# Contributions to Practical Laryngoscopy

**Published:** 1871-11

**Authors:** 


*Contributions to Practical Laryngoscopy.* Second series. By A. Ruppaner, A. M., M.D.

This contribution consists of an interesting case describing the removal of a large fibrous polypus, in the larynx, by thyrotomy, preceded by laryngo-tracheotomy. In connection with the clinical history of the case, it contains a colored plate with four figures. Fig. 1 showing the polypus in the larynx as seen in the laryngeal mirror before operation. Fig. 2 the same after removal by thyrotomy, and laid open in the median line, from apex to base. Fig. 3, appearance of the interior of the larynx six weeks after the operation. Fig. 4, appearance of the patient two months after the operation of thyrotomy, and four months after the operation of laryngo-tracheotomy. The canula still worn. The author promises the further observation in regard to it and its final result.

